# Development of a Non-Linear Bi-Directional Vortex-Induced Piezoelectric Energy Harvester with Magnetic Interaction

**DOI:** 10.3390/s21072299

**Published:** 2021-03-25

**Authors:** Wei-Jiun Su, Zong-Siang Wang

**Affiliations:** Department of Mechanical Engineering, National Taiwan University, No. 1, Sec. 4, Roosevelt Rd., Taipei 10617, Taiwan; r07522601@ntu.edu.tw

**Keywords:** piezoelectric energy harvester, bi-directional, vortex-induced vibration, magnetic interaction, nonlinearity

## Abstract

In this study, magnetic force is introduced to the design of a bi-directional U-shaped piezoelectric energy harvester for vortex-induced vibrations. The theoretical model of the beam structure is derived based on the Euler–Bernoulli beam theory. The vortex-induced vibration and the non-linear magnetic force are modeled according to the Rayleigh oscillator and the charge model, respectively. A prototype is fabricated and tested in two orthogonal directions under vortex-induced vibrations in a wind tunnel. Up and down wind-speed sweeps are carried out to investigate the non-linear responses of the harvester. The distance between the magnets and the length of the side beams are adjusted to examine the influence of the magnetic force on the lock-in region and voltage output of the harvester. Overall, the harvester shows strong non-linearity in the horizontal excitations. After adding magnets to the system, significant improvement of the lock-in region and the peak voltage is noticed in the horizontal mode under both up and down sweeps.

## 1. Introduction

Cantilevered piezoelectric energy harvesters (PEHs) have been extensively studied in the past two decades. Due to their narrow bandwidth and single-directional sensitivity, many studies have been carried out to overcome these issues. Some studies focus on designing multi-modal structures to improve the bandwidth [1,2]. Qi et al. [1] combined several piezoelectric cantilevered beams with different resonant frequencies to expand the bandwidth. Erturk et al. [2] designed a L-shaped piezoelectric beam to make the first two resonant frequencies close to improve the bandwidth. Non-linear force has also been widely used for bandwidth enhancement. By adding non-linear force into the system, the frequency response becomes non-linear so the bandwidth and power output can be improved under certain circumstances. Mechanical stoppers and magnetic force are the most commonly used techniques to introduce non-linear force to PEHs. Liu et al. [3] introduced mechanical stoppers on either one side or two sides of a cantilevered PEH. It was shown that the harvesting bandwidth was greatly expanded. Zhao et al. [4] used an incremental harmonic balance method to theoretically analyze the performance of a cantilevered PEH with a stopper. Significant improvement of harvesting bandwidth brought by the stopper was observed theoretically and experimentally. On the other hand, Stanton et al. [5] proposed a bi-stable PEH by adding a pair of magnets to a PEH to achieve broadband harvesting and improved the peak voltage under up sweeps. Lan and Qin [6] enhanced the performance of a bi-stable PEH by decreasing the potential barrier so the snap-through could be triggered even when the excitation was weak. Shih and Su [7] added magnets to a bi-directional U-shaped PEH to improve the bandwidth and voltage output.

Instead of scavenging kinetic energy from base excitations, many studies focus on harvesting flow energy, which includes fluid energy [8,9,10,11] and wind energy [12,13,14,15,16,17,18,19,20,21,22,23,24,25,26,27,28,29]. Wind-induced vibrations can be classified into galloping [12,13,14,15,16], flutter [17,18,19,20], wake galloping [21,22,23,24], and vortex-induced vibrations (VIVs) [25,26,27,28,29]. VIV-based energy harvesting is known for its large oscillations in the lock-in region and, therefore, attracts the most attention. Akaydin et al. [25] proposed a VIV-based PEH by attaching a cylinder to a piezoelectric cantilevered beam to acquire wind energy via VIVs. Zhou and Wang [26] arranged a pair of VIV-based cantilevered PEHs in a row to enhance the vortex and improve the harvesting efficiency. Zhang et al. [27] introduced magnetic force to the design of a VIV-based uni-directional PEH to improve its performance. The experimental results showed that the magnetic force caused the softening effect and enhanced the synchronization region and power output. Wang et al. [28] combined an upper piezoelectric beam and a bottom piezoelectric beam as a cross-coupled dual-beam PEH to achieve bi-directional harvesting from VIVs. Su and Lin [29] proposed a VIV-based U-shaped PEH for bi-directional harvesting. This PEH was capable of harvesting wind energy in two orthogonal directions of wind. The aspect ratio of the U-shaped structure could be adjusted to change the lock-in wind speed.

In the environment, wind may come from different directions. Therefore, the harvested energy will be improved if a PEH can scavenge wind energy from multiple directions. It was shown in previous works [5,6,7,27] that non-linear magnetic force effectively enhances the performance of PEHs. More specifically, magnetic force broadens the bandwidth of base-excitation type PEHs [5,6,7] and the lock-in region of VIV-based PEHs [27]. Therefore, we propose a design based on the bi-directional U-shaped PEH [29] with magnetic force introduced to improve the performance of the PEH. The theoretical model of the proposed VIV-based PEH was developed and validated with experiments. Up and down sweeps were carried out to examine the non-linear responses of the proposed PEH. This paper is arranged as follows. Section 2 introduces the design of the proposed VIV-based PEH and the theoretical model is derived in detail. Section 3 presents the experimental setup. The simulated and experimental results are illustrated and compared in Section 4. The conclusions are drawn in Section 5.

## 2. Design and Modeling

**Figure 1:**
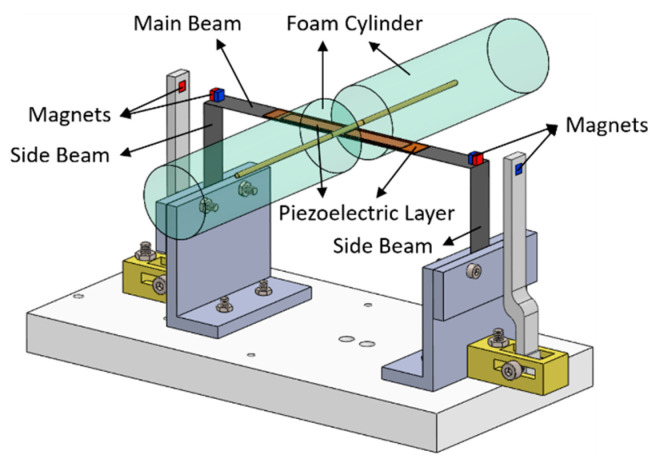
Schematic of the proposed bi-directional vortex-induced vibration (VIV) piezoelectric energy harvester (PEH).

**Figure 2:**
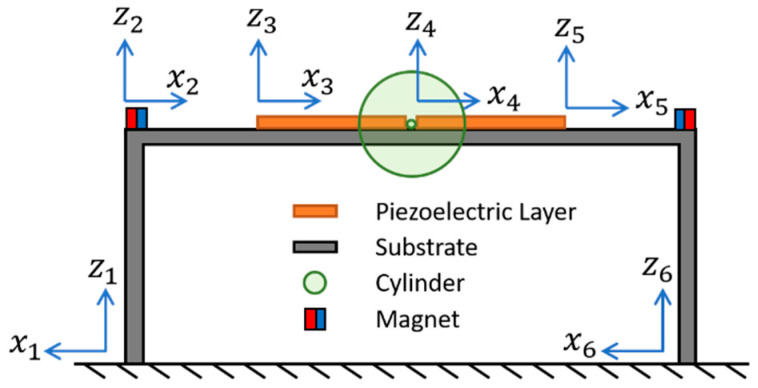
The local coordinates of the bi-directional PEH with magnetic interaction.

As shown in Figure 1, the proposed PEH is composed of a U-shaped beam, a pair of piezoelectric layers, a foam cylinder, and two pairs of magnets. The U-shaped beam structure comprises of a main beam and two side beams, which are fixed on the base. The piezoelectric layers are attached on the main beam symmetrically. The cylinder is attached to the center of the main beam to trigger VIVs. Two pairs of magnets are used to introduced non-linear repulsive force on the side beams. The PEH is modeled as a six-segment beam structure. The local coordinates of the PEH is depicted in Figure 2. The cylinder and the two magnets on the beams are assumed as point mass in the theoretical model.

### 2.1. Modeling of the Proposed Piezoelectric Energy Harvester (PEH)

The U-shaped beam is developed based on Euler-Bernoulli beam theory. While ignoring the damping, the undamped free vibration of the beam can be expressed as:

(1) $$\begin{array}{cc} YI_{n}\frac{\partial^{4}w_{n}(x,t)}{\partial x^{4}}+m_{n}\frac{\partial^{2}w_{n}(x,t)}{\partial t^{2}}=0 & n=1,\ldots ,6 \end{array}$$

where *YI* is the bending stiffness of the beam; *m* is the density of the beam per unit length; *w* is the transverse displacement and the subscript *n* denotes the segment number of the beam. The transverse displacement can be further expressed as:

(2) $$w_{n}(x_{n},t)=\sum_{r=1}^{\infty }\phi_{rn}(x_{n})\eta_{r}(t)$$

where *ϕ_r_* is the mode shape function; *η_r_* is the temporal function and the subscript *r* represents the mode. The model shape function can be written as:

(3) $$\phi_{rn}(x_{n})=A_{rn}\cos\left(\frac{\lambda_{rn}}{L_{n}}x_{n}\right)+B_{rn}\sin\left(\frac{\lambda_{rn}}{L_{n}}x_{n}\right)+C_{rn}\cosh\left(\frac{\lambda_{rn}}{L_{n}}x_{n}\right)+D_{rn}\sinh\left(\frac{\lambda_{rn}}{L_{n}}x_{n}\right)$$

where *L_n_* is the segment length; *A_rn_*, *B_rn_*, *C_rn_*, and *D_rn_* will be determined by the boundary and continuous conditions; *λ_rn_* is the eigenvalue and its relationship with the undamped natural frequency *ω_r_* can be represented as:

(4) $$\omega_{r}=\lambda_{rn}{}^{2}\sqrt{\frac{YI_{n}}{m_{n}L_{n}{}^{4}}}$$

The boundary and continuous conditions of the proposed PEH are listed in Equations (5)–(28):

(5) $$w_{r1}(0,t)=0$$

(6) $$w_{r1}{}^{\prime }(0,t)=0$$

(7) $$w_{r2}(0,t)=0$$

(8) $$w_{r5}(L_{5},t)=0$$

(9) $$w_{r6}(0,t)=0$$

(10) $$w_{r6}{}^{\prime }(0,t)=0$$

(11) $$w_{r1}{}^{\prime }(L_{1},t)=w_{r2}{}^{\prime }(0,t)$$

(12) $$YI_{1}w_{r1}{}^{\prime \prime }(L_{1},t)=YI_{2}w_{r2}{}^{\prime \prime }(0,t)$$

(13) $$w_{r2}(L_{2},t)=w_{r3}(0,t)$$

(14) $$w_{r2}{}^{\prime }(L_{2},t)=w_{r3}{}^{\prime }(0,t)$$

(15) $$YI_{2}w_{r2}{}^{\prime \prime }(L_{2},t)=YI_{3}w_{r3}{}^{\prime \prime }(0,t)$$

(16) $$YI_{2}w_{r2}{}^{\prime \prime \prime }(L_{2},t)=YI_{3}w_{r3}{}^{\prime \prime \prime }(0,t)$$

(17) $$w_{r3}(L_{3},t)=w_{r4}(0,t)$$

(18) $$w_{r3}{}^{\prime }(L_{3},t)=w_{r4}{}^{\prime }(0,t)$$

(19) $$YI_{3}w_{r3}{}^{\prime \prime }(L_{3},t)=YI_{4}w_{r4}{}^{\prime \prime }(0,t)$$

(20) $$YI_{3}w_{r3}{}^{\prime \prime \prime }(L_{3},t)=M_{mid}{\ddot{w}}_{r3}(L_{3},t)+YI_{4}w_{r4}{}^{\prime \prime \prime }(0,t)$$

(21) $$w_{r4}(L_{4},t)=w_{r5}(0,t)$$

(22) $$w_{r4}{}^{\prime }(L_{2},t)=w_{r5}{}^{\prime }(0,t)$$

(23) $$YI_{4}w_{r4}{}^{\prime \prime }(L_{4},t)=YI_{5}w_{r5}{}^{\prime \prime }(0,t)$$

(24) $$YI_{4}w_{r4}{}^{\prime \prime \prime }(L_{4},t)=YI_{5}w_{r5}{}^{\prime \prime \prime }(0,t)$$

(25) $$w_{r5}{}^{\prime }(L_{5},t)=-w_{r6}{}^{\prime }(L_{6},t)$$

(26) $$YI_{5}w_{r5}{}^{\prime \prime }(L_{5},t)=YI_{6}w_{r6}{}^{\prime \prime }(L_{6},t)$$

(27) $$w_{r1}(L_{1},t)=w_{r6}(L_{6},t)$$

(28) $$YI_{1}w_{r1}{}^{\prime \prime \prime }(L_{1},t)+YI_{6}w_{r6}{}^{\prime \prime \prime }(L_{6},t)=\left(\sum_{n=2}^{5}(m_{n}L_{n})+M_{t}\right){\ddot{w}}_{r1}(L_{1},t)$$

where *M_mid_* is the mass of the cylinder; *M_t_* is the total mass of the cylinder and the magnets attached on the beam. Equations (5)–(28) can be rearranged in the form of matrix as Equation (29):

(29) $$M\cdot \left[\begin{array}{c} A_{r1}\\ \vdots \\ D_{r6} \end{array}\right]=\left[\begin{array}{c} 0\\ \vdots \\ 0 \end{array}\right]$$

where *M* is a 24-by-24 matrix. The matrix *M* can be used to obtain the natural frequencies by making the determinant of *M* equal to zero. The mode shape function is mass-normalized according to Equation (30).

(30) $$\sum_{n=1}^{6}m_{n}\int_{0}^{L_{n}}\phi_{sn}\phi_{rn}dx+M_{mid}\phi_{s3}(L_{3})\phi_{r3}(L_{3})+\left(\sum_{n=2}^{5}(m_{n}L_{n})+M_{t}\right)\phi_{s1}(L_{1})\phi_{r1}(L_{1})=\delta_{rs}$$

Finally, the damping, excitation force and electromechanical coupling are considered in the model and the equation of motion of the PEH can be rewritten as:

(31) $${\ddot{\eta }}_{r}(t)+2\zeta_{r}\omega_{r}{\dot{\eta }}_{r}(t)+\omega_{r}{}^{2}\eta_{r}(t)+Q_{vs}v(t)=f(t)$$

where *ζ_r_* is the mechanical damping ratio and *f* is the normalized excitation force. *Q_vs_* is the electro-mechanical coupling coefficient and can be represented as:

(32) $$Q_{vs}=\theta_{s}\left[{\frac{d\phi_{r3}(x)}{dx}|}_{0}^{L_{3}}+{\frac{d\phi_{r4}(x)}{dx}|}_{0}^{L_{4}}\right]\;\;\;,where\;\;\theta_{s}=-\frac{E_{p}d_{31}b_{p}}{4h_{p}}(h_{d}{}^{2}-h_{c}{}^{2}),$$

*E_p_*, *b_p_*, *h_p_*, and *d_31_* are the young’s modulus, width, thickness and piezoelectric constant of the piezoelectric layer; *h_c_* is the distance from the neutral axis of beam to the bottom surface of the piezoelectric layer; *h_d_* is the distance from the neutral axis of beam to the top surface of the piezoelectric layer.

**Figure 3:**
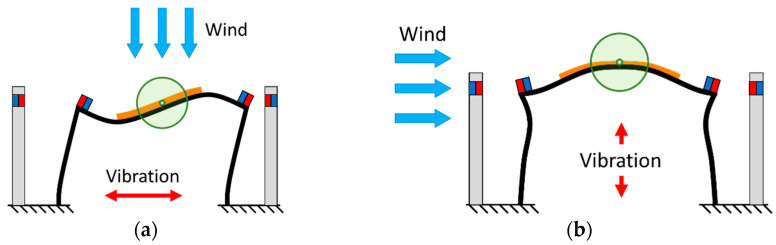
Mode shapes of the proposed bi-directional PEH under VIVs: (**a**) horizontal mode (**b**) vertical mode.

**Figure 4:**
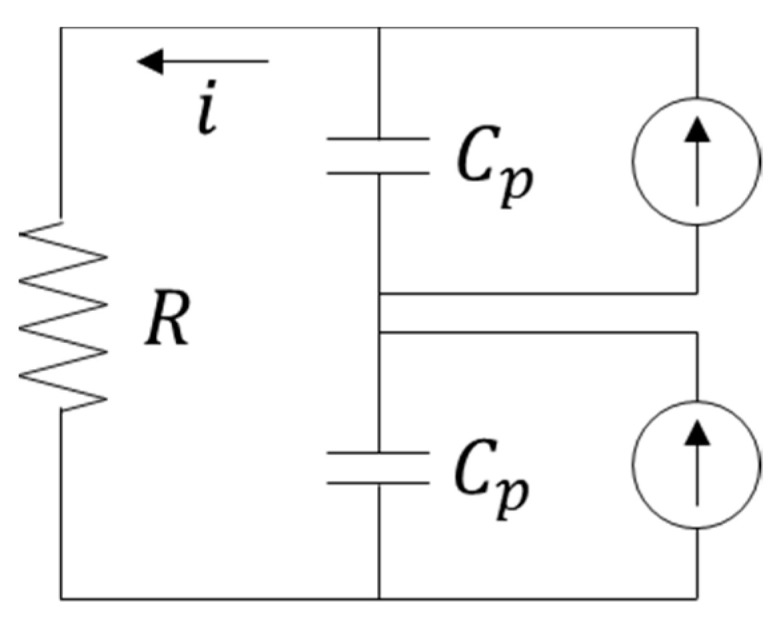
The equivalent circuit of the proposed PEH.

The mode shapes of the proposed PEH under excitations in the horizontal and vertical directions can be found in Figure 3. The mode is defined according to the vibration direction of the beam. The proposed PEH contains a pair of piezoelectric plates, which are connected in series. The equivalent circuit is shown in Figure 4. The equation of the circuit can be represented as:

(33) $$\frac{v(t)}{R}+C_{ps}\frac{dv(t)}{dt}+\kappa_{rs}{\dot{\eta }}_{r}=0$$

where *v* is the output voltage, *R* is the load resistance, *C_ps_* is equivalent capacitance of the two piezoelectric plates connected in series and *κ_rs_* is the electromechanical coupling coefficient, which can be written as:

(34) $$\kappa_{rs}=-Q_{vs}=-\theta_{s}\left[{\frac{d\phi_{r3}(x)}{dx}|}_{0}^{L_{3}}+{\frac{d\phi_{r4}(x)}{dx}|}_{0}^{L_{4}}\right]$$

**Figure 5:**
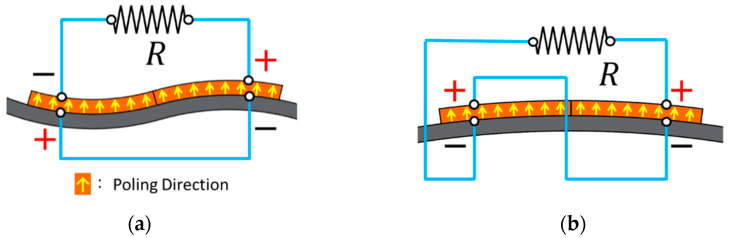
Circuit connections of the piezoelectric patches for (**a**) horizontal mode (**b**) vertical mode.

It can be found in Figure 3 that different modes result in different bending direction of the piezoelectric plates. Therefore, the two different modes required two different connection configurations to prevent voltage cancellation. The connection configurations are depicted in Figure 5.

### 2.2. Vortex-Induced Vibrations

In this study, the wake oscillator was based on the Rayleigh oscillators [30] and can be expressed as:

(35) $$\ddot{q}+\beta \omega_{s}({\left(\frac{\dot{q}}{\omega_{s}}\right)}^{2}-1)\dot{q}+\omega_{s}{}^{2}q=\frac{K}{D_{c}}\ddot{w}$$

where *β* is the viscosity coefficient for the structure, *K* is the coupling coefficient of the vortex and cylinder oscillation. It is noted that *β* and *K* are obtained via experimental results. *D_c_* is the diameter of the cylinder and *q* is the non-dimensional wake variable that describes the lifting in the near-wake region:

(36) $$q=2\frac{C_{L}}{C_{L_{0}}}$$

where *C_L_* is the lifting coefficient of a moving structure and *C_L_*_0_ is that of a stationary structure. *ω*_s_ is the vortex-shedding frequency and can be expressed as:

(37) $$\omega_{s}=\frac{2\pi StU_{w}}{D_{c}}$$

where *St* is Strouhal number, which is related to Reynolds number [31]. *U_w_* is the wind velocity.

**Figure 6:**
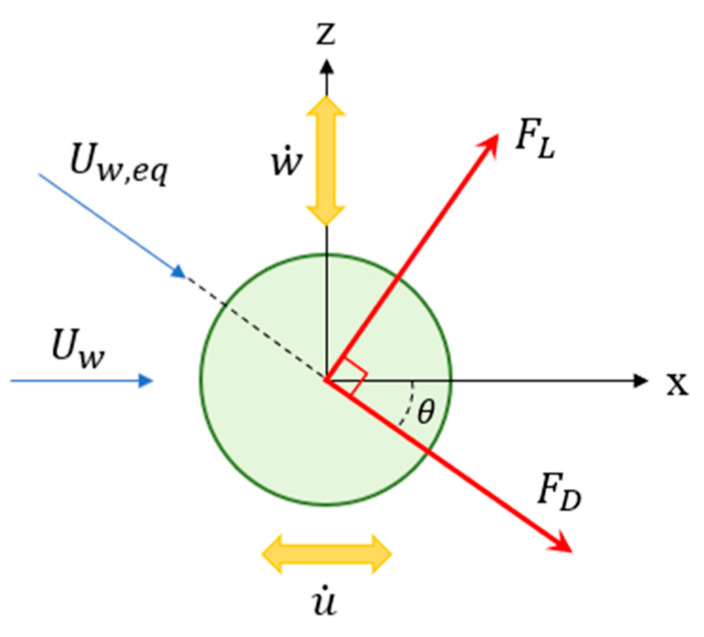
The local coordinate of the cylinder.

The local coordinate of the cylinder in vortex-induced vibrations is depicted in Figure 6. It is shown that when the wind of velocity *U_w_* flows over the cylinder, the cylinder will vibrate in the *x* direction with velocity of $\dot{u}$ and in the *y* direction with velocity of $\dot{w}$. Therefore, the equivalent wind velocity can be written as:

(38) $$U_{w,eq}=\sqrt{{\left(U_{w}-\dot{u}\right)}^{2}+{\dot{w}}^{2}}$$

The lift and drag force can then be written as [31]:

(39) $$F_{L}=\frac{1}{2}\rho_{a}U_{w,eq}{}^{2}D_{c}L_{c}C_{L}$$

(40) $$F_{D}=\frac{1}{2}\rho_{a}U_{w,eq}{}^{2}D_{c}L_{c}C_{D}$$

where *L_c_* is the length of the cylinder and *C_D_* is the drag coefficient of the moving cylinder. In vortex-induced vibrations, the vibration of the cylinder in the horizontal direction is much smaller than that in the vertical direction. Therefore, the displacement of the cylinder in the *x* direction is ignored and the force applied on the cylinder can be expressed as:

(41) $$F_{wz}=\frac{1}{2}\rho_{a}D_{c}L_{c}\left[C_{L}\cdot U_{w,eq}\cdot (U_{w}-\dot{u})-C_{D}\cdot U_{w,eq}\cdot \dot{w}\right]$$

where *ρ_a_* is the density of air. Because the velocity of the cylinder is much smaller than the wind speed, Equation (41) is rewritten as:

(42) $$F_{wz}=\frac{1}{2}\rho_{a}D_{c}L_{c}\left(C_{L}U_{w}{}^{2}-C_{D}U_{w}\dot{w}\right)$$

### 2.3. Magnetic Force

**Figure 7:**
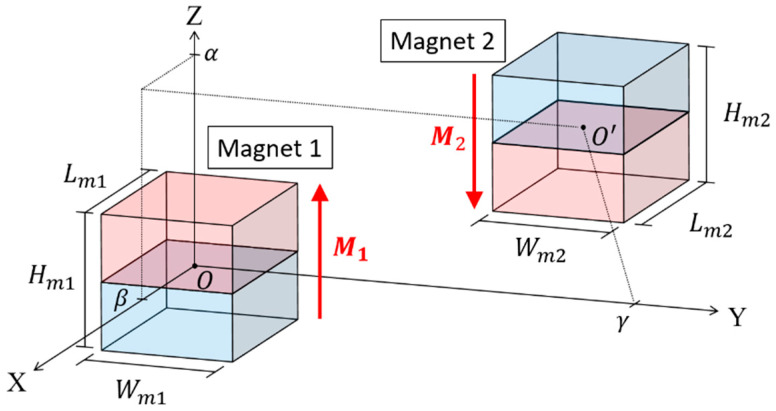
Schematic of a pair of cubic magnets.

In this section, the repulsive force of the cubic magnets is derived. The model was developed based on Furlani’s model [32] of rectangular magnets. The schematic of a pair of cubic magnets is illustrated in Figure 7. Based on the charge model, the surface charge density can be expressed as:

(43) $$\sigma_{i}=\vec{M_{i}}\cdot \hat{n}$$

where the subscript *i* is used to indicate the magnet number; *n* is the surface normal; *M_i_* is the magnetization and can be further represented as:

(44) $$M_{i}=\frac{Br_{i}}{\mu_{0}}$$

where *Br_i_* is the residual induction and *μ*_0_ is the vacuum permeability.

Consider two cubic magnets that carry charges of *Q_m_*_1_ and *Q_m_*_2_. The potential energy caused by a charge *dQ_mi_* of magnet *i* can be expressed as:

(45) $$d\varphi_{mi}=\frac{dQ_{mi}}{4\pi }\frac{1}{R_{m}}$$

where *R_m_* is the distance between the charge and an arbitrary point of a magnet. The total potential energy caused by a surface of magnet *i* on a charge can be written as:

(46) $$\varphi_{mi}=\oint_{A_{mi}}d\varphi_{mi}dA_{mi}$$

where *A_mi_* is the surface area. The external magnetic field can then be represented as:

(47) $$H^{ext}(x,y,z)=-\nabla \varphi_{m1}(x,y,z)$$

The magnet flux density is expressed as:

(48) $$\begin{array}{ll} B^{ext}(x,y,z) & =\mu_{0}\times H^{ext}(x,y,z)\\ & =B_{x}^{ext}(x,y,z)\cdot \hat{x}+B_{y}^{ext}(x,y,z)\cdot \hat{y}+B_{z}^{ext}(x,y,z)\cdot \hat{z} \end{array}$$

where the *x*, *y*, and *z* components can be written as:

(49) $$\left\{ \begin{array}{l} B_{x}^{ext}(x,y,z)=\frac{\mu_{0}M_{1}}{4\pi }\sum_{i=1}^{2}\sum_{j=1}^{2}\sum_{k=1}^{2}{\left(-1\right)}^{i+j+k}\times \ln\left[(y-y_{j})+r\right]\\ B_{y}^{ext}(x,y,z)=\frac{\mu_{0}M_{1}}{4\pi }\sum_{i=1}^{2}\sum_{j=1}^{2}\sum_{k=1}^{2}{\left(-1\right)}^{i+j+k}\times \ln\left[(x-x_{i})+r\right]\\ B_{z}^{ext}(x,y,z)=-\frac{\mu_{0}M_{1}}{4\pi }\sum_{i=1}^{2}\sum_{j=1}^{2}\sum_{k=1}^{2}{\left(-1\right)}^{i+j+k}\times \tan^{-1}\left[\frac{\left(x-x_{i})(y-y_{j}\right)}{(z-z_{k})r}\right] \end{array}\right.$$

where

(50) $$r=\sqrt{{\left(x-x_{i}\right)}^{2}+{\left(y-y_{j}\right)}^{2}+{\left(z-z_{k}\right)}^{2}}$$

The variables *x_i_*, *y_j_*, and *z_k_* represent the coordinates of the boundaries of the magnets. The force between magnet 1 and a charge on magnet 2 can be expressed as:

(51) $$dF_{m}=B^{ext}(x,y,z)dQ_{m2}$$

Due to the materials, temperature, humidity etc., the real permeability *μ* will differ from vacuum permeability *μ_0_*. By replacing the vacuum permeability with real permeability, the magnetic force can be expressed as:

(52) $$F_{x,y,z}=-\frac{\mu M_{1}M_{2}}{4\pi }\sum_{i=1}^{2}\sum_{j=1}^{2}\sum_{k=1}^{2}\sum_{l=1}^{2}\sum_{m=1}^{2}\sum_{n=1}^{2}{\left(-1\right)}^{i+j+k+l+m+n}\times f_{x,y,z}(u,v,w,s)$$

where

(53) $$\left\{ \begin{array}{l} f_{x}=v\left\{u-u\ln(v+s)-w\tan^{-1}(\frac{u}{w})+w\tan^{-1}(\frac{uv}{ws})\right\}\;\;+\frac{1}{2}us+\frac{w^{2}-v^{2}}{2}\ln(u+s)\\ f_{y}=u\left\{v-v\ln(u+s)-w\tan^{-1}(\frac{v}{w})+w\tan^{-1}(\frac{uv}{ws})\right\}\;\;+\frac{1}{2}vs+\frac{w^{2}-u^{2}}{2}\ln(v+s)\\ f_{z}=-ws+real\left(uw\coth^{-1}(\frac{u}{s})+vw\coth^{-1}(\frac{v}{s})\right)+uv\tan^{-1}(\frac{uv}{ws}) \end{array}\right.$$

and

(54) $$\left\{ \begin{array}{l} u=\alpha +{\left(-1\right)}^{l}\frac{L_{m2}}{2}-{\left(-1\right)}^{i}\frac{L_{m1}}{2}\\ v=\beta +{\left(-1\right)}^{m}\frac{W_{m2}}{2}-{\left(-1\right)}^{j}\frac{W_{m1}}{2}\\ w=\gamma +{\left(-1\right)}^{n}\frac{H_{m2}}{2}-{\left(-1\right)}^{k}\frac{H_{m1}}{2}\\ s=\sqrt{u^{2}+v^{2}+w^{2}} \end{array}\right.$$

### 2.4. Model of the Proposed Non-Linear Bi-Directional PEH

In this section, we will summarize the structural, VIV, and magnet models derived in the previous sections to develop the complete model of the proposed bi-directional PEH. Referring to Equation (31), the external force *f_wz_* caused by VIV can be expressed with respect to the mode of the PEH:

(55) $$f_{wz}(t)=\left\{ \begin{array}{l} \mathrm{Horizontal:}\;\frac{1}{2}\rho_{a}D_{c}L_{c}\left(C_{L}U_{w}{}^{2}-C_{D}U_{w}\phi_{r1}(L_{1}){\dot{\eta }}_{r}\right)\phi_{r1}(L_{1})\\ \mathrm{Vertical:}\;\frac{1}{2}\rho_{a}D_{c}L_{c}\left(C_{L}U_{w}{}^{2}-C_{D}U_{w}\phi_{r3}(L_{3}){\dot{\eta }}_{r}\right)\phi_{r3}(L_{3}) \end{array}\right.$$

**Figure 8:**
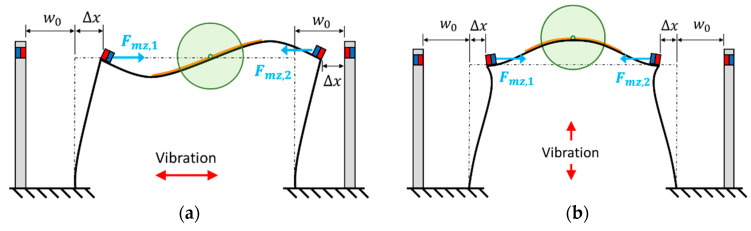
Schematics of the PEH with magnets in (**a**) the horizontal mode, (**b**) the vertical mode.

The magnetic force is then integrated in the model. The schematics of the proposed PEH with magnets are shown in Figure 8. Two pairs of magnets are installed symmetrically on the PEH to generate repulsive force. The magnetic force can be represented as:

(56) $$f_{mz}(t)=\left\{ \begin{array}{l} \mathrm{Horizontal:}\;\phi_{r1}(L_{1})F_{mz,1}(w_{0}+\phi_{r1}(L_{1})\eta_{r}(t))-\phi_{r6}(L_{6})F_{mz,2}(w_{0}-\phi_{r6}(L_{6})\eta_{r}(t))\\ \mathrm{Vertical:}\;\phi_{r1}(L_{1})F_{mz,1}(w_{0}+\phi_{r1}(L_{1})\eta_{r}(t))-\phi_{r6}(L_{6})F_{mz,2}(w_{0}+\phi_{r6}(L_{6})\eta_{r}(t)) \end{array}\right.$$

Therefore, the complete governing equations of the proposed bi-directional PEH can be represented as:

(57) $$\{\begin{array}{l} {\ddot{\eta }}_{r}(t)+2\zeta_{r}\omega_{r}{\dot{\eta }}_{r}(t)+\omega_{r}{}^{2}\eta_{r}(t)+Q_{vc}v(t)=f_{wz}(t)+f_{mz}(t)\\ \frac{v_{c}(t)}{R}+C_{pc}\frac{dv_{c}(t)}{dt}+\kappa_{rc}{\dot{\eta }}_{r}(t)=0\\ \ddot{q}+\beta \omega_{s}({\left(\frac{\dot{q}}{\omega_{s}}\right)}^{2}-1)\dot{q}+\omega_{s}{}^{2}q=\frac{K}{D_{c}}{\ddot{w}}_{m}(t) \end{array}$$

where ${\ddot{w}}_{m}(t)$ is the acceleration of the cylinder and can be expressed as:

(58) $${\ddot{w}}_{m}(t)=\left\{ \begin{array}{l} \mathrm{Horizontal:}\;{\ddot{w}}_{1}(L_{1},t)\\ \mathrm{Vertical:}\;{\ddot{w}}_{3}(L_{3},t) \end{array}\right.$$

## 3. Experiment

**Figure 9:**
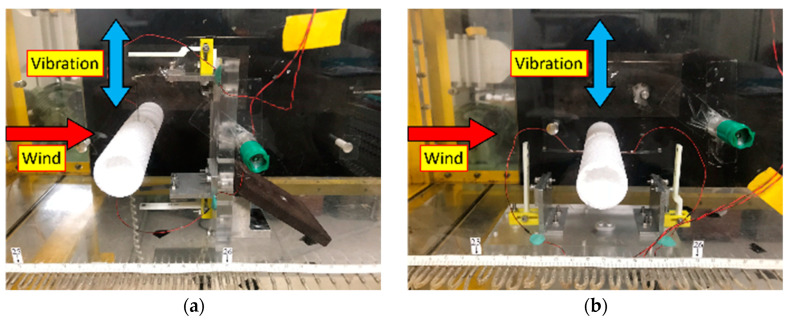
Setup of the proposed PEH installed in the wind tunnel for (**a**) horizontal mode, and (**b**) vertical mode.

The prototype of the proposed PEH installed in a wind tunnel is shown in Figure 9. The PEH was installed in two different orientations in order to receive wind from two different directions. It can be seen in the supplementary videos 1 and 2 that the vibration direction of the beam structure is perpendicular to the wind direction. The U-shaped structure was a stainless steel (SUS301) beam bent to a shape of U. A pair of macro fiber composite (MFC) patches (M-2807-P2) were attached symmetrically on the main beam of the U-shaped structure. A foam cylinder was installed at the middle of the main beam to induce vortex. The parameters of the prototype are listed in Table 1.

**Figure 10:**
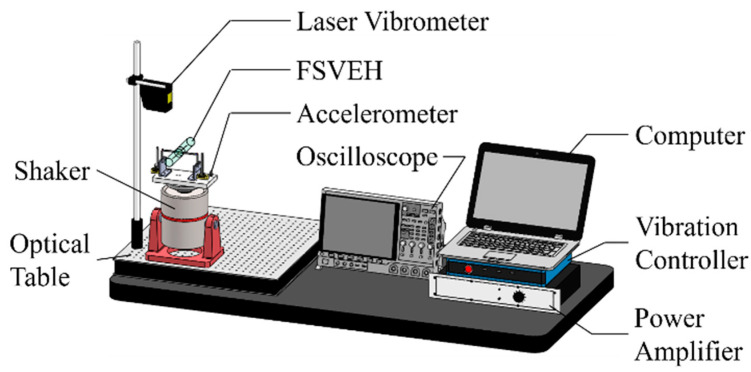
Schematic of the platform for base-excitation tests.

**Figure 11:**
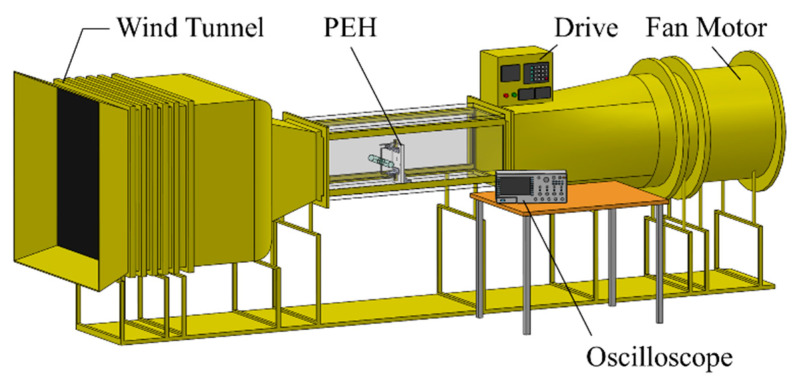
Schematic of the wind tunnel for VIV tests.

Figure 10 shows the platform for base excitations. The PEH was installed on a shaker (LDS-V406), which was controlled and driven by a vibration controller (UCON VT-9002) and a power amplifier (LDS PA-100E). An accelerometer (PCB 352C66) was used to detect the acceleration of the shaker and send a feedback signal to the controller for closed-loop control. The displacement of the PEH was acquired by a laser displacement sensor (Mti LTS-120-40) and the voltage output was obtained by an oscilloscope (Keysight DSOX4042A). The damping ratio was obtained in the base excitation tests with the acceleration of 0.05 G. The PEH was then tested in a wind tunnel (LonGwin LW-9300S) for VIVs. The schematic of the wind tunnel tests can be seen in Figure 11.

## 4. Results

In this section, the proposed PEH was firstly examined under base excitations to validate the resonant frequency and fit the damping ratio. The proposed PEH was then investigated in a wind tunnel for VIV tests. The simulated results were compared with the experimental results. Different magnet distances and side-beam lengths were tested to understand their influence on the performance of the PEH.

### 4.1. Proposed PEH under Base Excitation

**Figure 12:**
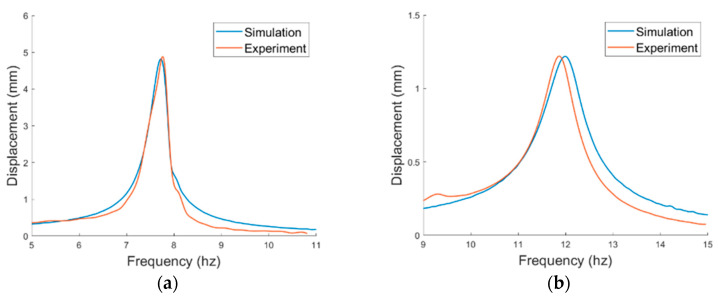
Displacement responses in short circuit under base excitations in (**a**) horizontal mode, (**b**) vertical mode.

**Figure 13:**
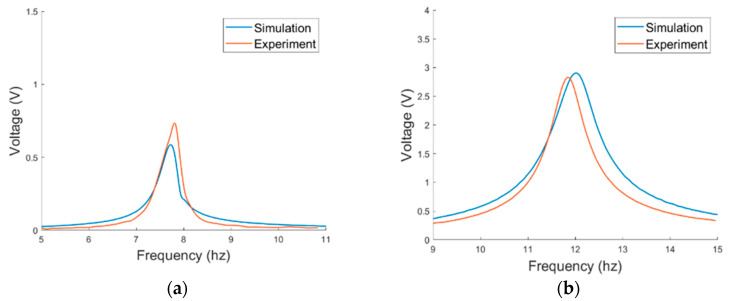
Voltage responses under base excitations in (**a**) horizontal mode, (**b**) vertical mode.

The prototype of the PEH was first tested under base excitations to verify the resonant frequencies. The displacement in short circuit is depicted in Figure 12. The displacement of the vertical mode was measured at the middle point of the main beam while that of the horizontal mode was measured at the free end of the side beam. It can be seen that the simulated resonant frequencies match the experimental ones well. The errors between the simulated and experimental resonant frequencies of the horizontal and vertical modes were 1.15% and 1.35%, respectively. The damping ratios were obtained by fitting the simulation displacement with the experimental displacement and are arranged in Table 2. The output voltage of the PEH in series connection is shown in Figure 13. A notable difference between the peak voltage of the simulated and experimental results in the horizontal mode may result from the imperfect symmetry of the two MFC patches. Therefore, the bending of the two MFC patches are not identical. Overall, the simulation and experimental results are well matched so the theoretical model will be further utilized for the simulation of the VIV-based PEH.

### 4.2. Proposed PEH under Vortex-Induced Vibrations

**Figure 14:**
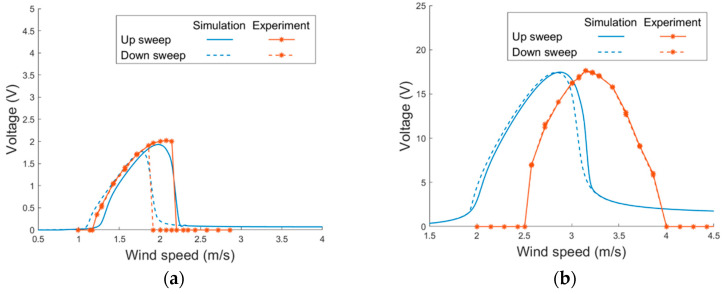
Voltage responses of the proposed PEH without magnets under VIVs in (**a**) horizontal mode (**b**) vertical mode.

The proposed PEH was then examined in the wind tunnel tests. As the system was non-linear, the voltage responses were examined under wind-speed sweeps to see how the nonlinearity influence the responses. The PEH without magnets was first tested. Figure 14 depicts the simulated and experimental voltage responses of the PEH. The viscosity coefficient *β* and vortex-related coupling coefficient *K* were obtained via experiments and are shown in Table 3. It can be seen that the model predicts well the responses of the horizontal mode in both up and down sweeps. The model is accurate enough even without taking the geometric non-linearity into consideration. The non-linearity caused by the VIVs demonstrates the hardening effect, which enlarges the lock-in region for up sweeps. On the other hand, the non-linear effect is not notable in the vertical mode. The responses in up and down sweeps are almost identical in both the simulation and experimental results. It can be noted that the experimental lock-in region is shifted to a higher region when compared with the simulation results. The mismatch may result from the deformation of the PEH by the wind pressure, which strengthens the stiffness of the structure and, therefore, increases the lock-in wind speed in experiments.

**Figure 15:**
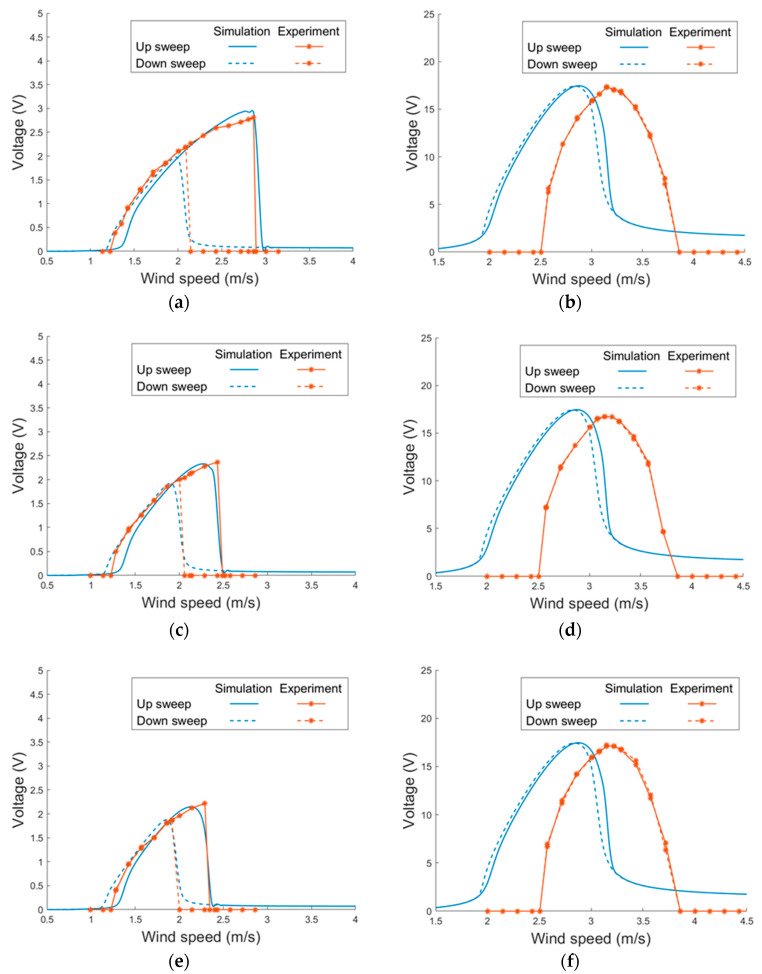
Voltage responses of the proposed PEH with different magnet spacing under VIVs (side-beam length = 40 mm). (**a**) 17 mm, horizontal (**b**) 17 mm, vertical (**c**) 19 mm, horizontal (**d**) 19 mm, vertical, (**e**) 21 mm, horizontal, (**f**) 21 mm, vertical.

Figure 15 depicts the voltage responses of the magnet-integrated PEH with different magnet spacing: 17 mm, 19 mm and 21 mm. Three settings of magnet spacing were examined in the simulation and the wind tunnel tests. It can be seen that the experimental results show a similar trend as the simulation results. In all the vertical modes of all the three magnet settings, the width of the lock-in region and the peak voltage of the theoretical and experimental results show a good agreement. In the horizontal mode, the theoretical model predicts the width of the lock-in region and peak voltage under both up and down sweeps well. Stronger non-linearity is noted in the horizontal mode when the magnet gap is smaller.

**Figure 16:**
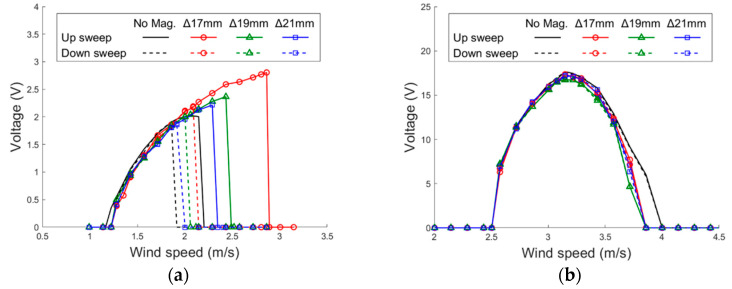
Experimental voltage responses of the proposed PEH with different magnet gaps under VIVs (side-beam length = 40 mm). (**a**) horizontal mode (**b**) vertical mode.

The experimental results of the PEH with different settings of magnet gap are arranged in Figure 16. It can be noted that the magnetic force improves the lock-in wind speed range and peak voltage in all the three settings of the horizontal mode. It can be seen that the magnet force influences the horizontal mode greatly but has little impact on the vertical mode. That is because the magnets are attached on the end of the side beams, where the displacement is almost zero in the vertical mode. The performance improvement is more significant as the magnet gap is reduced. The PEH demonstrates better performance in up sweeps than down sweeps because of the hardening effect. Moreover, it is noted that the magnet-integrated PEH outperforms that without magnets not only in the up sweeps but also in the down sweeps. In the setting with the gap of 17 mm, which shows the most significant improvement among all settings, the lock-in region is widened by 34.8% and the peak voltage is enhanced by 33.2% in the up sweeps. In the down sweeps, significant improvement of both the peak voltage and lock-in region can still be seen. Overall, adding magnets to the design of the U-shaped PEH improve peak voltage and usable wind-speed region in both up and down sweeps.

**Figure 17:**
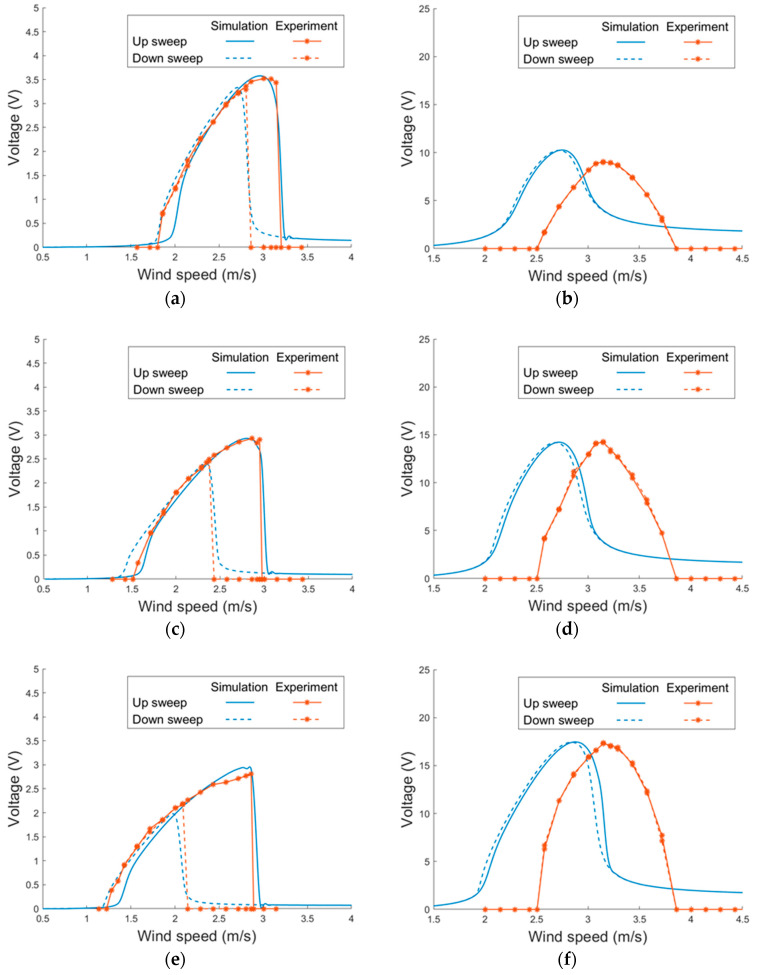
Voltage responses of the proposed PEH with different side-beam lengths under VIVs. (magnet gap = 17 mm) (**a**) 30 mm, horizontal (**b**) 30 mm, vertical (**c**) 35 mm, horizontal (**d**) 35 mm, vertical, (**e**) 40 mm, horizontal, (**f**) 40 mm, vertical.

**Figure 18:**
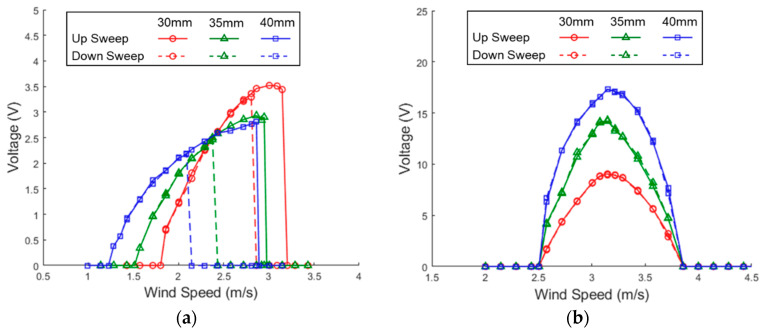
Experimental voltage responses of the proposed PEH with side-beam lengths under VIVs (magnet gap = 17 mm). (**a**) horizontal mode (**b**) vertical mode.

Figure 17 shows the simulation and experimental results of the PEH with different side-beam lengths. To change the length of the side beams in experiments, we used the same prototype of the PEH but clamped the side beams at different locations to keep all the parameters unchanged except the side-beam length. The side-beam length has a strong impact on the horizontal mode but only a slight impact on the vertical mode. As the length of the side beam increases, the stiffness of the PEH decreases so the resonant frequency as well as the lock-in wind speed are shifted to a lower region. It can be seen in the simulation and experimental results that lock-in wind speed region of the PEH with the shortest side beams in the horizontal mode during up sweeps is significantly higher than that of the two configurations with longer side beams. The lock-in region in the vertical mode shows no significant difference among the three configurations. However, it can be found that the peak voltage of the vertical mode is improved when the side beams are extended because the mode shape changes. The experimental results of the PEH with different length of the side beams are rearranged in Figure 18. It can be found that the responses in the horizontal mode of all the three configurations show a hardening effect during the wind-speed sweeps. The hardening effect becomes more significant as the length of the side beam increases due to the decrease of the stiffness of the PEH in the horizontal mode.

It can be noted in Figure 16 and Figure 18 that both extending the side beams and reducing the magnet gap can enhance the hardening effect and, therefore, enlarge the lock-in region. However, the side-beam length not only alters the lock-in region but also the peak voltage of both horizontal and vertical modes while the magnet gap only impacts the lock-in region. Moreover, extending the side beams slightly improve the lock-in region in up sweeps. No significant impact on the lock-in region is seen in down sweeps. Nevertheless, reducing the magnet gap demonstrates an improved lock-in region in both up and down sweeps.

## 5. Conclusions

In this study, we proposed a magnet-integrated VIV-based PEH to achieve bi-directional wind-energy harvesting, as wind comes from various directions in the environment. Magnetic force is introduced to the PEH in order to enhance its performance. The theoretical model is derived based on Euler–Bernoulli beam theory for the structure, Rayleigh oscillators for the VIVs, and the charge model for the magnetic force. A prototype was fabricated and first examined under base excitations to obtain the damping ratio of the PEH. The PEH was then tested in a wind tunnel for VIVs and the VIV-related coupling coefficients were acquired. Finally, the PEH was integrated with magnets attached at the ends of the side beams and examined in VIV tests. Magnet gap and side-beam length were both examined to see their influence on the performance. Wind-speed up and down sweeps were conducted to observe the non-linearity of the proposed PEH. In both up and down sweeps, it was shown that the magnet force enhanced the lock-in region and the peak voltage of the horizontal mode in both up and down sweeps but had little impact on the vertical mode. The PEH with the smallest magnet gap, which was 17 mm, exhibited the best performance among the three settings. On the other hand, when the magnet gap was fixed at 17 mm, shortening the side beams could only enhance the lock-in region of up sweeps but not that of down sweeps. Moreover, the peak voltage of both modes were altered greatly. The study demonstrates that magnetic force can significantly improve the peak voltage and usable wind-speed range in the horizontal mode under both up and down sweeps without influencing the vertical mode.

## Figures and Tables

**Table 1:** Parameters of the prototype.

Parameters	Symbol	Value
Length of the main beam		130 mm
Length of the side beam		40 mm
Length of the magnet on the PEH	*L_mag,_*_1_	3 mm
Length of the magnet on the base	*L_mag,_*_2_	4 mm
Length of the epoxy layer	*L_ex_*	33.5 mm
Length of the macro fiber composite (MFC) patch	*L_p_*	33.5 mm
Length of the cylinder	*L_c_*	200 mm
Young’s modulus of the substrate	*Y_s_*	178 GPa
Young’s modulus of the epoxy	*Y_ex_*	27 MPa
Young’s modulus of the MFC patch	*Y_e_*	30.336 GPa
Thickness of the substrate	*h_s_*	0.07 mm
Thickness of the epoxy layer	*h_ex_*	0.02 mm
Thickness of the MFC patch	*h_p_*	0.3 mm
Width of the substrate	*b_s_*	12.7 mm
Width of the epoxy layer	*b_ex_*	6.4 mm
Width of the MFC patch	*b_p_*	6.4 mm
Density of the substrate	*ρ_s_*	7800 kg/m^3^
Density of the epoxy	*ρ_e_*	1200 kg/m^3^
Density of the MFC patch	*ρ_p_*	5440 kg/m^3^
Weight of the magnet on the PEH	*M_mag_*	0.255 g
Magnetization	*Br*	1.32 T
Relative permeability	*μ_r_*	1.2
Permeability	*μ_0_*	4*π* × 10^−7^ N∙A^−2^
Diameter of the cylinder	*D_c_*	40 mm
Weight of the cylinder	*M_c_*	2.7 g
Weight of the stick	*M_stick_*	0.3 g
Capacitance (MFC)	*C_p_*	21.2 nF
Piezoelectric constant (MFC)	*d*_31_	−250 pm/V
Load resistance (base excitation)	*R*	500 kΩ
Load resistance (VIVs)	*R*	1 MΩ

**Table 2:** Damping ratio.

Mode	Damping Ratio
horizontal	0.0182
vertical	0.0326

**Table 3:** Fitted parameters of the proposed PEH under VIVs.

Mode	*K*	*β*
horizontal	17.7	0.12
vertical	17	0.24

## Data Availability

Not applicable.

## Supplementary Materials

The following are available online at https://www.mdpi.com/1424-8220/21/7/2299/s1. Video S1: Vortex-induced vibration of the PEH in the horizontal mode, Video S2: Vortex-induced vibration of the PEH in the vertical mode.
